# Testing the recent theories for the origin of the hermaphrodite flower by comparison of the transcriptomes of gymnosperms and angiosperms

**DOI:** 10.1186/1471-2148-10-240

**Published:** 2010-08-03

**Authors:** Raquel Tavares, Mathilde Cagnon, Ioan Negrutiu, Dominque Mouchiroud

**Affiliations:** 1 Université de Lyon, F-69622, Lyon, France; Université de Lyon 1, Villeurbanne; CNRS, UMR5558, Laboratoire Biométrie et Biologie Évolutive, Villeurbanne, France; 2 Université de Lyon, F-69622, Lyon, France; École Normale Supérieure de Lyon, Lyon; Laboratoire de Reproduction et Développement des Plantes, Lyon, France

## Abstract

**Background:**

Different theories for the origin of the angiosperm hermaphrodite flower make different predictions concerning the overlap between the genes expressed in the male and female cones of gymnosperms and the genes expressed in the hermaphrodite flower of angiosperms. The Mostly Male (MM) theory predicts that, of genes expressed primarily in male versus female gymnosperm cones, an excess of male orthologs will be expressed in flowers, excluding ovules, while Out Of Male (OOM) and Out Of Female (OOF) theories predict no such excess.

**Results:**

In this paper, we tested these predictions by comparing the transcriptomes of three gymnosperms (*Ginkgo biloba*, *Welwitschia mirabilis *and *Zamia fisheri*) and two angiosperms (*Arabidopsis thaliana *and *Oryza sativa*), using EST data. We found that the proportion of orthologous genes expressed in the reproductive organs of the gymnosperms and in the angiosperms flower is significantly higher than the proportion of orthologous genes expressed in the reproductive organs of the gymnosperms and in the angiosperms vegetative tissues, which shows that the approach is correct. However, we detected no significant differences between the proportion of gymnosperm orthologous genes expressed in the male cone and in the angiosperms flower and the proportion of gymnosperm orthologous genes expressed in the female cone and in the angiosperms flower.

**Conclusions:**

These results do not support the MM theory prediction of an excess of male gymnosperm genes expressed in the hermaphrodite flower of the angiosperms and seem to support the OOM/OOF theories. However, other explanations can be given for the 1:1 ratio that we found. More abundant and more specific (namely carpel and ovule) expression data should be produced in order to further test these theories.

## Background

In spite of the great and ever growing amount of morphological and molecular data accumulating from paleobotany, phylogenetics and evo-devo analysis, the origin of the angiosperms hermaphrodite flower is still the "abominable mistery" Charles Darwin referred to, in a letter written to the British botanist Joseph Dalton Hooker, 150 years ago [1-3].

Many theories have been proposed to explain angiosperm origins, differing on the features proposed for the ancestor of the flower and on the evolutionary mechanisms giving rise to the "modern" hermaphrodite structure. The most recent ones have the advantage that they can be tested using molecular data from extant plants, namely gymnosperms and angiosperms [4]. One of these recent hypotheses, the "Mostly Male Theory", suggests that the angiosperms flower derives from the male reproductive structures of the ancestor, on which ectopic ovules (normally located on the female axis) have developed. The male unit would thus become bisexual and later some microsporophylls (modified leaves bearing the male structures producing microspores) would have enclosed the ovules, giving rise to the angiosperms carpel [5]. Alternatively, the Out Of Male and the Out of Female theories propose that homeotic changes in gene expression would have given rise to a perianth-less flower-like structure (a flower where the reproductive structures are not protected, for instance by petals or sepals) with male reproductive units in the basal region and female reproductive units in the apical region [6,4].

These theories make different predictions concerning the overlap between the genes expressed in the male and female cones of gymnosperms and the genes expressed in the hermaphrodite flower of angiosperms. Since it states that the ancestral flower derived from a mainly male axis (on which only the ovules would be female) the Mostly Male (MM) Theory predicts an excess of orthologs of gymnosperm male genes expressed in the angiosperms flower. In other words, of the genes expressed in the hermaphrodite flower, "more should have close homologs (or orthologs, if gene trees are sufficiently resolved to demonstrate orthology) active in male gymnosperm reproductive structures rather than in female structures » [5]. On the other hand, proposing a "mixed" structure, half male half female, as the flower ancestor, the Out Of Male (OOM) and the Out Of Female (OOF) theories predict no excess of gymnosperm male (or female) cone genes expressed in the angiosperm flower [7].

The Floral Genome Project [8] has generated abundant sequence collections of several gymnosperm and angiosperm species. Large EST datasets, capturing thousands of sequences of genes expressed during flower development in each species, have been accumulated.

Using both data from the FGP and from the Arabidopsis [9] and rice genome projects [10,11], we tested these predictions by comparing the transcriptomes of three gymnosperms (*Ginkgo biloba*, *Welwitschia mirabilis *and *Zamia fisheri*) and two angiosperms (*Arabidopsis thaliana *and *Oryza sativa*). We chose Arabidopsis and rice for the abundance of EST data and because their complete genome is sequenced, which ensures that the "right" angiosperm ortholog is found for each gymnosperm gene. We used all gymnosperm species for which we had enough EST data from male and female cones' libraries. This is unfortunately not the case for *Pinus taeda*, from which not such libraries exist, despite the total of more than 300 000 EST sequences available from this species.

## Methods

### Coding sequences data

cDNA and peptide sequences of the angiosperms were retrieved from the Rice Genome Annotation Project http://rice.plantbiology.msu.edu/ release 5-Jan 2007-66 710 sequences) [10,11] and from The Arabidopsis Information Resource (TAIR) http://www.arabidopsis.org/ TAIR8 release - April 2008-38 963 sequences) [9]. Unigene sequences of the gymnosperms were retrieved from The Floral Genome Project http://fgp.bio.psu.edu/*Welwitschia mirabilis*: 6 214 unigenes and *Zamia fischeri*: 9 967 unigenes) [8] and the New York Plant Genomics Consortium http://nypgenomics.org/*Ginkgo biloba*: 3 820 unigenes made by Brenner and collaborators [12]).

### Expression data

We used total of 1 526 133 EST from *Arabidopsis thaliana*, 1 220 876 EST from *Oryza sativa*, 10 129 EST from *Welwitschia mirabilis*, 8 252 EST from *Zamia fischeri *and 6 250 EST from *Ginkgo biloba *(dbEST release 080108 GenBank, NCBI). EST libraries obtained from mRNA extractions of the same organs, types of libraries, developmental stages and physiological conditions (called here expression categories) were grouped, according to the annotations provided by the NCBI (library.report). We generated complete expression data for the 66 710 rice cDNA and for the 38 963 Arabidopsis cDNA, using all the ESTs from a total of 317 rice libraries (grouped in 51 expression categories) and 181 Arabidopsis libraries (39 expression categories). In order to perform our tests, we then selected the expression data from flowers and inflorescences (angiosperms), male and female (early developmental stages) cones (gymnosperms) and vegetative tissues (leaves and pools of vegetative organs). Table 1 summarizes the information of the EST numbers and libraries used in these comparisons.

**Table 1:** Number of ESTs per library used in the reproductive/vegetative and male/female comparisons.

**EST data**					
**Arabidopsis**		**Rice**			
			
**ORGANS**	**EST nber**	**ORGANS**	**EST nber**		
flower (4 lib)	**8551**	fl+pan (44 lib)	**256423**		
flower+infl (8 lib)	**9927**	leaf (20 lib)	**105113**		
leaf+rosette (8 lib)	**2913**	veg pool (2 lib)	**30398**		
veg pool (2 lib)	**2501**				
TOTAL EST nber	**23892**	TOTAL EST nber	**391934**		
	
**Ginkgo**		**Welwitschia**		**Zamia**	
		
ORGANS	EST nber	ORGANS	EST nber	ORGANS	EST nber
female cone (1 lib)	**2117**	female strob (1 lib)	**5283**	megasp+ovu (1 lib)	**3711**
male cone (1 lib)	**2047**	microsp (2 lib)	**4846**	microsp (1 lib)	**4541**
female leaf (1 lib)	**1061**				
male leaf (1 lib)	**1025**				
TOTAL EST nber	**6250**	TOTAL EST nber	**10129**	TOTAL EST nber	**8252**

nber: number; lib: library(ies); infl: inflorescence; veg: vegetative; fl: flower; pan: panicle; strob: strobili; megasp: megasporophyll; microsp: microsporopyll; ovu: ovule.

Expression data within each species was obtained by linking the EST to the cDNA (or unigenes) of the species. This was made by blastn between the cDNA (unigenes) sequences (query) and the EST sequences (bank). A threshold alignment score of E-10 was used to filter the results, and only the alignments of at least 100 bp and with a minimum of 95% of identity between the sequences were retained. Multiple hits of the same EST to one cDNA sequence were discarded (only the best was retained) and whenever the same EST aligned with different cDNA, we kept only the hits having a score of at least 90% of the score of the best hit. One "good" hit (respecting the criteria described above) with an EST was sufficient for a gene to be considered expressed in the organ corresponding to the EST library. No quantitative analysis of the expression level was performed, only the presence/absence of each gene in the different EST libraries was considered.

### Orthology

The orthology between the angiosperm and gymnosperm genes was established by the best reciprocal hit method. This means that a blast is performed between the two species genes using each species both as the query and as the subject for the alignments. The best hit for each query sequence is retained, the results of the two (reciprocal) blast are compared, and only the pairs of sequences corresponding to a best hit in both blast are retained as pairs of orthologs.

Blast was performed between the peptide sequences mentioned above for Arabidopsis and rice and either peptide (*Ginkgo biloba*) or nucleotide (all three gymnosperms) sequences of the gymnosperms. The peptide sequences of *G.biloba *were obtained applying a polypeptide prediction pipeline, prot4EST [13] to the unigene sequences. For this species, orthology with Arabidopsis and rice was established by reciprocal best hit using both blastp between the peptide sequences of the two species or blastx and tblastn between the peptide sequences of Arabidopsis and rice and the nucleotide sequences of *G.biloba*. As the results were very similar, orthologs between the other two gymnosperms and Arabidopsis and rice were determined by reciprocal best hit using blastx and tblastn between the peptide sequences of Arabidopsis and rice and the nucleotide sequences of the gymnosperms (*i.e*. no peptide sequence predictions were used for the gymnosperms). All blast results were filtered with an E-10 threshold for the alignment score.

### Biological tests

The test of the gene expression predictions of the theories for the origin of the hermaphrodite flower was done by comparing the number of genes expressed both in the male cone of the gymnosperms and in the angiosperm flower with the number of genes expressed both in the female cone of the gymnosperms and in the angiosperm flower. It is thus a male/female comparison. We did not compare the absolute number of genes, this value has instead been "normalized" by the number of genes expressed in each cone (and thus eliminating the influence of the size of the EST library) or, more precisely, by the number of genes expressed in each cone for which we were able to find an ortholog in the angiosperm species.

A consequence of the relative small size of the gymnosperm EST libraries is that we most certainly do not detect all the genes actually expressed in each tissue. This means that we may not use this type of data to detect tissue specific genes (any false negative in a tissue expression set would produce a false positive in another tissue specific expression set). We thus made the analysis using the total set of genes expressed in each male or female cone of the gymnosperms, independently of its expression in the other, female or male, cone.

In order to evaluate the suitability of the expression EST data for this kind of test, we made other comparisons, which worked as a control of the main analysis. One strong hypothesis that legitimates Frohlich's and Parker's predictions [5] is that differences in the tissue identity (which, in this case, is used as an indicator of the tissue origin) can be evaluated at a transcriptomics level by comparing proportions of expressed genes. If this is true, we should expect, for instance, the proportion of genes expressed in common in the reproductive organs of a gymnosperm and the flower of an angiosperm to be greater than the proportion of genes expressed in common in the reproductive organs of a gymnosperm and the vegetative organs of an angiosperm. We tested this by comparing the proportion of genes having an angiosperm ortholog and being expressed in each gymnosperm male and female cones and in a reproductive organ of the angiosperms with the proportion of genes having an angiosperm ortholog and being expressed in each gymnosperm male and female cones and in a vegetative organ of the angiosperms. Two kinds of pools of reproductive organs (flower and flower and inflorescences) and of vegetative organs (leaves and pools of vegetative organs) were used in the analysis. We did this for the three gymnosperms and the two angiosperms, making a total of 36 "control" comparisons.

### Statistical analysis

The statistical evaluation of the expression ratios was made by 1) estimating the confidence interval (CI) of the proportion of the gymnosperm genes (with an angiosperm ortholog) expressed in a gymnosperm organ, from which the angiosperm orthologs are expressed in an angiosperm organ. The choice of using a CI estimation of a frequency instead of the frequency itself was made because the total n (number of orthologs found between the two species that are expressed in a gymnosperm organ) is not the same for the different proportions calculated; 2) calculating the ratio between two of these CI (for instance, between the CI of the expression in a gymnosperm male cone and an angiosperm flower versus the CI of the expression in a gymnosperm female cone and an angiosperm flower, for the main test of the analysis). This was done by dividing the lower limit of one CI (ex CI A) by the upper limit of the other (CI B) and, inversely, by dividing the upper limit of the CI A by the lower limit of CI B. We thus obtained a CI for the ratio (CI A/B = ]minA/maxB; maxA/minB[) and then calculated the mean value of this CI; 3) testing the null hypothesis of this ratio being equal to 1 (the p-values were calculated). These analyses were performed with R [14].

## Results

Table 2 shows the number of unigenes of each gymnosperm that are expressed in each gymnosperm EST library, and the fraction of those for which an angiosperm ortholog could be found. The number of genes expressed in a gymnosperm organ (male or female) for which the angiosperm ortholog is detected in the angiosperms flower EST libraries is also shown.

**Table 2:** Number of sequences used, with expression and orthology information.

			**Arabidopsis**		**Oryza**	
			
			**cDNA/pep**	**EST**	**cDNA/pep**	**EST**
			38963	1526133	66710	1220876
**number expressed in:**	**EST**	**unigenes**	**orthologs**	**orth exp flower**	**orthologs**	**orth exp flower**
**Gb male cones**	2047	1481	892	326	884	645
**Gb female cones**	2117	1330	718	245	707	515
**Wm male cones**	4846	3325	2089	498	2096	1283
**Wm female cones**	5283	3705	2847	640	2840	1792
**Zf male cones**	4541	3032	325	8	917	34
**Zf female cones**	3711	4021	1525	377	1622	937

Gb: *Ginkgo biloba*; Wm: *Welwitschia mirabilis*; Zf: *Zamia fisheri*; pep: peptides; orth exp flower: orthologs expressed in the flower.

### Problem with the *Zamia fisheri* male bank

The number of angiosperm orthologs found among the *Zamia fisheri *male expressed unigenes was abnormally low, especially for Arabidopsis (10%, i.e. approximately 4 times less than the proportion found for the female expressed genes). Most of the unigenes for which no angiosperm ortholog was found either had no similarity with any GenBank sequence or corresponded to transposable elements sequences. The number of genes with an ortholog detected in the angiosperms flower libraries was, as a consequence, also very low, which probably explains the "atypical" results found for the *Z. fisheri *male expressed genes comparisons.

The results of the male/female comparisons and of the main control tests are shown in table 3.

**Table 3:** Ratios and p-values of the male/female and reproductive/vegetative tissues comparisons.

		**Arabidopsis**		**Oryza**	
			
		**ratio**	**p-value**	**ratio**	**p-value**
**Ginkgo**	**male/female**	**1,089**	**0,292**	**1,01**	**1**
	rep female/veg	4,87	7,64E-38	1,64	1,83E-26
	rep male/veg	4,912	4,51E-52	1,79	2,94E-41
	rep female/leaf	2,58	4,74E-21	1,3	2,24E-10
	rep male/leaf	3,13	2,42E-36	1,28	1,11E-11
					
**Welwitschia**	**male/female**	**1,087**	**0,17**	**0,97**	**0,186**
	rep female/veg	3,84	1,58E-70	2,05	1,03E-130
	rep male/veg	3,93	1,14E-56	1,89	3,10E-77
	rep female/leaf	2,277	7,23E-37	1,32	4,86E-31
	rep male/leaf	2,252	1,23E-28	1,29	2,90E-18
					
**Zamia**	**male/female**	**0,178**	**7,69E-13**	**0,07**	**3,60E-159**
	rep female/veg	4,198	1,79E-45	1,87	1,31E-52
	rep male/veg	4,898	0,574	3,93	0,003
	rep female/leaf	2,667	1,78E-28	1,3	2,11E-13
	rep male/leaf	1,187	0,371	1,29	1

rep: reproductive; veg: vegetative organs pool; leaf: leaves.

### Control test

All the reproductive/vegetative comparisons showed a significant excess of genes expressed in common in the male or female cones of a gymnosperm and in the flower of an angiosperm, compared to the genes expressed in common in the male or female cones of a gymnosperm and in the vegetative organs (leaves or pools of vegetative organs) of the angiosperms. The ratios varied from 1.2 (*Z. fisheri *male expressed genes, but see previous section) to 4.9 (*G. biloba *male expressed genes) times more genes in common between the reproductive organs of the two species than between the reproductive organs of one species and the vegetative organs of the other. This excess was found for the comparisons of the three gymnosperms with the two angiosperms and using both the angiosperm flower EST libraries alone (data not shown) or a pool of angiosperm flower and inflorescence EST libraries (shown on the table), and using both the angiosperm leaves EST libraries or the angiosperm vegetative organs pool EST libraries. All the p-values are highly significant (from E-10 to E-130), except the ones of the *Zamia fisheri *male comparisons (but see previous section).

### Test of the theories of the origin of the flower

None of the gymnosperm male/female comparisons showed the excess of gymnosperm male cone genes expressed in the angiosperm flower predicted by the Mostly Male Theory for the origin of the flower. Except for the *Zamia fisheri *comparisons, where a significant excess of female cone expressed genes was detected among the genes expressed in the Arabidopsis or rice flower (but see first section of the Results), all the male/female ratios were not significantly different from 1 (ratios from 0.97 to 1.09; p-values > 0.15). The results were very similar, for each gymnosperm, when using Arabidopsis or rice as the angiosperm species.

## Discussion

Our results indicate equivalent proportions of gymnosperm male and female organs genes expressed in the angiosperm hermaphrodite flower. This 1:1 ratio is not in agreement with the Mostly Male Theory prediction of an excess of male gymnosperm genes expressed in the flower of the angiosperms. We can think of different explanations for this non-detection of differences between male and female genes.

A first explanation would be that the OOM/OOF theories are correct. This would be in agreement with the expression studies by Vásquez-Lobo and collaborators [15] on gymnosperm LEAFY-like genes that aimed to further test an important observation for the MM theory: of the two LEAFY-like genes in gymnosperms, only one paralog was kept in angiosperms, and this paralog seemed to have a male-specific expression pattern [1]. These analysis do not find male vs. female-specific expression for the two LEAFY-paralogs in several gymnosperm species, and thus do not support distinctive functions of the two LEAFY-like genes in specifying male and female reproductive organs, which is compatible with the OOM/OOF theories but not with the MM one. However, it should be emphasized that while the MM theory predicts a significant difference between the proportions of the two types of expressed genes (which we were able to test and not able to find) the corresponding predictions of the OOM and OOF theories, i.e. a "significant equivalence" of the proportions of expressed genes, may not be strictly tested. In other words, our work does not falsify the OOM/OOF theories, but it is not able to verify them.

The absence of differences may also mean that expression divergence between gymnosperms and angiosperms is too great to allow these kind of comparisons. Whole genome duplications in the angiosperm lineage, in particular, could contribute to this expression divergence because of neo or subfunctionalisation of gene duplicates orthologous to male or female gymnosperm single genes. Nonetheless, our control experiment revealed that the proportion of orthologous genes expressed in the reproductive organs (both male and female) of the gymnosperms and in the angiosperms flower is significantly higher than the proportion of orthologous genes expressed in the reproductive organs of the gymnosperms and in the angiosperms vegetative tissues. This was found for all the species and libraries tested, and is not what we expect only by chance. The results of the control experiment thus seem to indicate that lack of signal in the data is not the right explanation for the 1:1 ratio.

One last possible explanation would be that female and male ancestral characteristics of the angiosperm flower may not be measured by the number of genes expressed in common with female and male tissues of the ancestor, but that differences between sexes are due to only a few genes or are quantitative, i.e. due to the level of expression of the (eventually the same) genes. The fact that this might be a real obstacle to the analysis is suggested by the relatively widespread capacity of stamen tissues to undergo feminization. The ectopic production of ovules, stigmatic tissues or valve-type tissues by stamens, or even their complete transformation into carpels (called pistillody) as is the case in some papaya genotypes [16], indicate that somatic tissues of the stamen can switch developmental programs locally and relatively late in development [17-19]. The genetic control of the process is not well understood, but work in wheat has showed that pistillody is caused in this species by alterations to the class-B MADS-box gene expression pattern in given lines following cytoplasmic substitution through recurrent backcrossing [20]. The fact that in the early diversification of the angiosperms stamen evolution has been viewed as more labile or changing more rapidly [21], further indicates that male-female structures may share a large set of common gene networks. An alternative explanation for the male/female shared expression patterns would concern genes expressed in the ovules. The prediction of the MM theory specifically excludes ovule-expressed gymnosperm (female) genes. It could be that enough ovule expressed genes have been retained (or redeployed) in the angiosperm flower to mask any differences among genes not derived from ovules.

Distinction between these alternative explanations would need quantitative expression data. The available EST data, comprising normalized libraries and libraries of relatively small size, do not allow us to make quantitative comparisons. Microarrays could provide such information - unfortunately such data are not available for gymnosperm reproductive structures.

Another interesting analysis would be to concentrate on the typical angiosperm flower feature, the organ for which the different theories propose a different origin - the carpel. For the MM theory, the carpel tissue has a male origin (except for the ovules), while the OOM and OOF theories propose an ancestral female identity for all the female tissues in the hermaphrodite angiosperm flower. We have used affymetrix microarray data from Arabidopsis http://affymetrix.arabidopsis.info/, including expression data from the carpel, and performed the same kind of comparisons between male and female (the main test) and reproductive and vegetative (the control experiment) gymnosperm genes (EST were used for the expression data of gymnosperms) expressed in the angiosperm flower. No coherent ratios of reproductive expression over vegetative expression were obtained in the control experiment, *i.e*. no excess of gymnosperm reproductive tissues genes over vegetative tissues genes was found to be expressed in the angiosperm flower. This means that we cannot trust the male/female expression ratios observed. Mixing EST and microarray expression data, with different thresholds of detection, is probably preventing the detection of any eventual significant signal.

## Conclusions

In conclusion, we have tested the MM theory prediction of gene expression comparison between gymnosperms and angiosperms reproductive structures using all the adequate data available up to now. Our results do not support the MM theory prediction, but further analysis, using quantitative and more detailed expression data (namely ovule and carpel angiosperm expressed genes and eventually male and female specific gymnosperm expressed genes) are needed to determine if the MM theory is, or is not, the correct explanation for the origin of the hermaphrodite flower.

## Authors' contributions

RT and MC analysed and interpreted the data. RT conceived and designed the project, wrote the manuscript and supervised the work. IN and DM helped in the conception of the study, the interpretation of data and the revision of the manuscript. All the authors have given final approval of the version to be published.
